# *Ziziphus budhensis* Seed Extracts: Phytoconstituents and Preliminary In Vitro and In Vivo Biological Activities

**DOI:** 10.1002/cbdv.202503447

**Published:** 2026-05-14

**Authors:** Samjhana Bharati, Binita Maharjan, Timila Shrestha, Gopal Gautam Khatri, Hari Prasad Devkota, Ram Lal Swagat Shrestha, Stefano Dall'Acqua

**Affiliations:** ^1^ Department of Chemistry Amrit Campus Tribhuvan University Kathmandu Nepal; ^2^ Department of Chemistry Trichandra Multiple Campus Tribhuvan University Kathmandu Nepal; ^3^ Graduate School of Pharmaceutical Sciences Kumamoto University Kumamoto Japan; ^4^ Department of Pharmaceutical and Pharmacological Sciences University of Padova Padova Italy

**Keywords:** bioactivities, in vitro, in vivo, *Ziziphus budhensis*

## Abstract

*Ziziphus budhensis* (Rhamnaceae) is an endemic Nepalese species with significant religious and economic importance in Nepal, mainly due to the use of its seeds in religious ornaments. Its edible fruits and woody seeds are used for preparing Bodhichitta mala. Despite their significance, the seeds have received little scientific attention, particularly regarding their chemical composition and biological properties. To address this gap, sequential extracts of *Z. budhensis* seeds were prepared using solvents of increasing polarity. Preliminary bioassays, namely antioxidant, antibacterial, and enzyme‐inhibitory activities, as well as toxicity studies, were performed. Biological screening showed moderate antioxidant and *α*‐amylase inhibitory activities, whereas noticeable antibacterial and antifungal effects were witnessed for acetone and ethyl acetate extracts. Brine shrimp lethality test and acute toxicity mouse models indicated limited or no toxicity for the different extracts. Liquid chromatography‐mass spectrometry allowed the identification of fatty acids, triterpenoids, phytosterols, cyclopeptide alkaloids, flavonoids, and other phenolic compounds. Overall, this study offers the first integrated chemical and biological insight into *Z*. *budhensis* seeds and points to their potential relevance as a natural source of antimicrobial bioactive compounds, cosmetic products, and skin health formulations.

## Graphical abstract

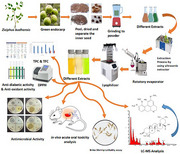

## 1 Introduction

Medicinal plants have long been invaluable sources of medicinal compounds, forming the foundation of herbal medicines and traditional remedies worldwide. A significant number of clinically used drugs originate from, or are inspired by, plant‐derived secondary metabolites. This underscores the continued relevance of natural products as invaluable reservoirs of chemically diverse structures with a wide variety of biological activities, which remain central to modern drug discovery and development. These metabolites, including phenolics, flavonoids, alkaloids, terpenoids, and glycosides, demonstrate a wide range of pharmacological activities, including antioxidant, antimicrobial, anti‐inflammatory, and enzyme‐inhibitory effects. The qualitative and quantitative profiles of these compounds vary significantly across plant species, organs, developmental stages, and environmental conditions, making endemic and underexplored plants desirable targets for phytochemical and biological investigations [1, 2, 3, 4].

The genus *Ziziphus Mill*. (Rhamnaceae) comprises 170–200 species of shrubs and small trees and is widely distributed across warm‐temperate and tropical regions. Several *Ziziphus* species bear edible fruits and have long been used in traditional medicine systems for the treatment of insomnia, anxiety, inflammation, gastrointestinal disorders, infections, and metabolic diseases. *Ziziphus* species are reported to have multiple therapeutic effects, including sedative, anxiolytic, hypnotic, antitumor, and anti‐inflammatory activities [5, 6, 7]. Phytochemical studies across the genus have demonstrated a rich diversity of bioactive constituents, including cyclopeptide alkaloids, triterpenoids, flavonoids, saponins, sterols, and phenolic acids. Many of these compounds have been linked to a broad field of pharmacological activities, such as sedative, antioxidant, antidiabetic, and antimicrobial effects [8].

Despite this growing body of literature, research within the *Ziziphus* remains uneven, with several species and plant parts still insufficiently explored. *Z. budhensis* is an endemic species of the Nepal Himalaya, commonly known as “Bodhichitta” or “Buddha Mala”. The species holds exceptional religious, cultural, and economic importance, as the woody endocarps of its fruits are traditionally carved into rosary beads used in Buddhist spiritual practices. The commercial value of *Z. budhensis* beads is determined mainly by their size, color, and number of facets, with high‐quality garlands fetching prices of several thousand USD [9, 10]. The species is predominantly cultivated in the Kavrepalanchok district of Nepal, where it plays an important role in agroforestry practices and supports local livelihoods. Botanically, *Z. budhensis* is a spiny, deciduous tree that flowers between March and April and bears fruits from May to August. The fruits are smooth, yellowish‐brown, and edible; however, they are seldom consumed because of their considerable economic value [11]. The woody endocarp is cartilaginous, comprising one to three locules that enclose one or two seeds. The seeds are often crafted into bracelets, lockets, and necklaces, serving more as fashion items than spiritual objects. Seeds with smaller golden seeds hold the highest price; a garland of 108 premium beads can cost up to 2000 USD [10, 12, 13]. Scientific investigations on *Z. budhensis* remain limited and have predominantly focused on taxonomy, ecology, propagation, and socioeconomic dimensions rather than medicinal chemistry. Early studies mainly reported its taxonomic identification and ecological characteristics [9], seed dormancy and germination behavior [12], as well as cultivation status and market potential in Nepal [10, 11]. While these works highlight the cultural and economic importance of the species, they offer minimal insight into its phytochemical profile or biological activities. Consequently, comprehensive phytochemical characterization and systematic pharmacological evaluation of *Z. budhensis* remain largely unexplored to date.

Notably, our recent investigation of *Z. budhensis* leaves identified a diverse variety of secondary metabolites, including phenolic compounds, cyclopeptide alkaloids, benzylisoquinoline alkaloids, triterpenoids, and saponins, and demonstrated significant in vitro and in vivo biological activities [13]. This study represents the first comprehensive chemical and biological characterization of a defined plant tissue from *Z. budhensis* and reveals pronounced tissue‐specific variation in metabolite profiles. Nevertheless, other plant parts, such as fruits, bark, and especially seeds, remain largely unexplored in terms of their phytochemical composition and bioactivity, despite their widespread traditional utilization.

Seeds of *Z. budhensis* are of particular interest, as they constitute the most economically valuable part of the plant and are widely used for religious and ornamental purposes. Despite their significance, there is not enough scientific information concerning their chemical composition, biological activities, and safety profile. To date, no comprehensive study has integrated sequential extraction, LC–MS‐based phytochemical profiling, in vitro bioactivity assessment, and in vivo toxicity evaluation of *Z. budhensis* seed extracts.

**FIGURE 1:**
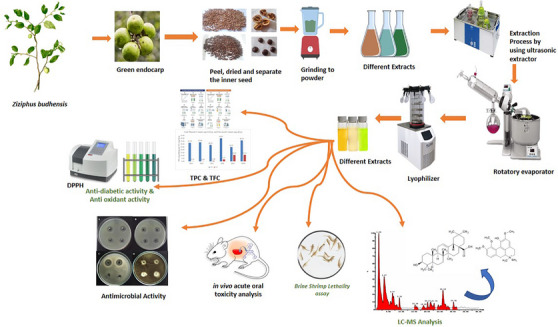
Overview of the research process.

To address this critical knowledge gap, the present study aims to: (i) characterize the phytochemical composition of *Z. budhensis* seed extracts using liquid chromatography–mass spectrometry (LC–MS), (ii) evaluate their preliminary in vitro biological activities, including antioxidant, antimicrobial, and *α*‐amylase inhibitory effects, and (iii) assess their safety through brine shrimp lethality and in vivo acute oral toxicity studies. By focusing on a previously unexplored plant part of an endemic and culturally significant species, this work seeks to elucidate the medicinal potential of *Z. budhensis* seeds and situate them within the broader scientific context of the genus *Ziziphus* (Figure 1).

## 2 Results and Discussion

### 2.1 Total Phenolic and Flavonoid Content Analysis

**FIGURE 2:**
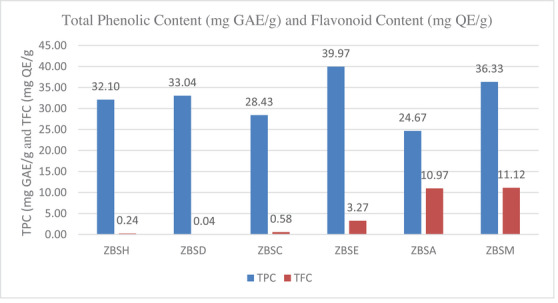
Total phenolic (TPC) and total Flavonoid Content (TFC) of the seed extracts of *Z. budhensis*. ZBSH: Hexane, ZBSD: Dichloromethane, ZBSC: Chloroform, ZBSE: Ethylacetate, ZBSA: Acetone, ZBSM: Methanol.

The total phenolic and flavonoid contents of seed extracts were determined using spectrophotometric methods. The total phenolic content (TPC) was measured as milligrams of gallic acid equivalent. Likewise, the total flavonoid content (TFC) per gram of dried material was stated in milligrams of quercetin equivalents [14, 15] (Figure 2).

Among the six different seed extracts, the ethyl acetate extract shows the highest value of phenolic content (39.97 mg GAE/g), and the methanol extract shows the highest value of flavonoid content (11.12 mg QE/g extract) in the seed extract. Phenolic compounds detected by the TFC assay can be extracted significantly with more hydrophilic solvents, whereas they are not extracted with hexane, dichloromethane, or chloroform. The TPC and TFC values of *Z. budhensis* seed extracts indicate the presence of phenolic compounds that may yield a positive response in the test, warranting further investigations to be conducted in the paper's proceedings using liquid chromatography and mass spectrometry.

### 2.2 Antibacterial and Antifungal Screening Analysis

The search for new bioactive compounds or extracts with antibacterial or antifungal activity can be valuable, given the need for new potential active ingredients. For this reason, *Z. budhensis* seed extracts were assessed for their antibacterial and antifungal effects. As a screening, we tested the extracts at 100 mg/mL. The two Gram‐positive bacteria (*Bacillus subtilis* ATCC 6051 and *Staphylococcus aureus* ATCC 6538P), the two Gram‐negative bacteria (*Escherichia coli* ATCC 8739 and *Klebsiella pneumoniae* ATCC 700603), and the fungus *Candida albicans* ATCC 2091 were chosen and completed the tests. The usefulness of the extracts was assessed by measuring the inhibition zones around the zones where the extracts were functional (Table 1).

**TABLE 1:** Antibacterial activity of seed extracts of *Z. budhensis*.

Bacterial/Fungal strain	Reference culture	Type	Positive control (c+) cm	Negative control (c‐) cm	ZBSH ZOI (cm)	ZBSD ZOI (cm)	ZBSC ZOI (cm)	ZBSE ZOI (cm)	ZBSA ZOI (cm)	ZBSM ZOI (cm)
*Escherichia coli*	ATCC 8739	Gram‐ve	2.5	0	1.1	1.2	1.1	2.3	2.7	1.7
*Klebsiella pneumoniae*	ATCC 700603	Gram‐ve	2.1	0	1.1	1.2	1.1	1.8	2.7	1.2
*Bacillus subtilis*	ATCC 6051	Gram+ve	2.8	0	1.1	1.2	1.2	1.8	2.7	1.5
*Staphylococcus aureus*	ATCC 6538P	Gram+ve	2.4	0	1.3	1.3	1.2	2.2	2.7	1.3
*Candida albicans*	ATCC 2091	Fungi	2.6	0	1.3	1.2	1.2	2.3	2.3	1.6

ZBSH: Hexane, ZBSD: Dichloromethane, ZBSC: Chloroform, ZBSE: Ethylacetate, ZBSA: Acetone, ZBSM: Methanol.

**FIGURE 3:**
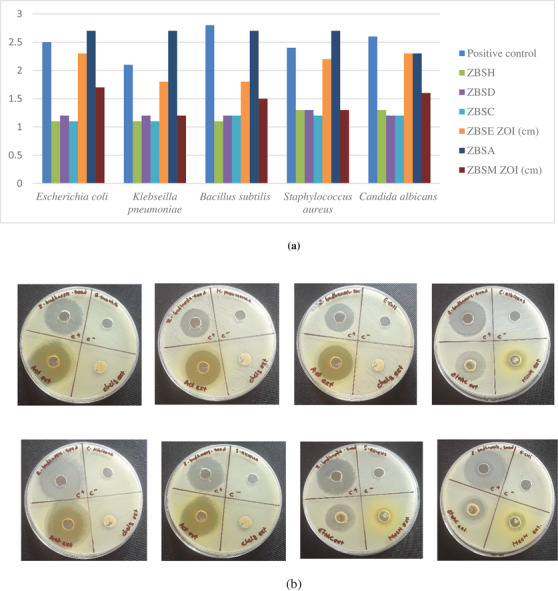
Antibacterial and antifungal screening analysis of seed extract with ZOI. (a): Antibacterial and antifungal screening analysis of seed extract. ZBSH: hexane, ZBSD: dichloromethane, ZBSC: chloroform, ZBSE: ethyl acetate, ZBSA: acetone, ZBSM: methanol; positive control: kanamycin. (b): Potential zone of inhibition of acetone extract and ethylacetate.

According to Figure 3a,b, the extracts displayed varying degrees of inhibition zones and showed some activity against the selected microorganism, with kanamycin used as the reference compound. Acetone and ethyl acetate show the most significant antibacterial and antifungal activity. In comparison, the acetone extract showed the maximum antibacterial activity against the tested bacteria, with the most effective against Gram‐negative *Escherichia coli* and *Klebsiella pneumoniae*, and Gram‐positive *Staphylococcus aureus*, producing a zone of inhibition (ZOI) similar to or higher than that of the positive control. This result indicates significant bioactivity of these extracts, underscoring the need for detailed chemical analysis to determine their composition and identify which classes of compounds present in seed extracts may inhibit bacterial growth, thereby making the extracts more valuable as natural antimicrobial agents.

### 2.3 Brine Shrimp Lethality Test

**FIGURE 4:**
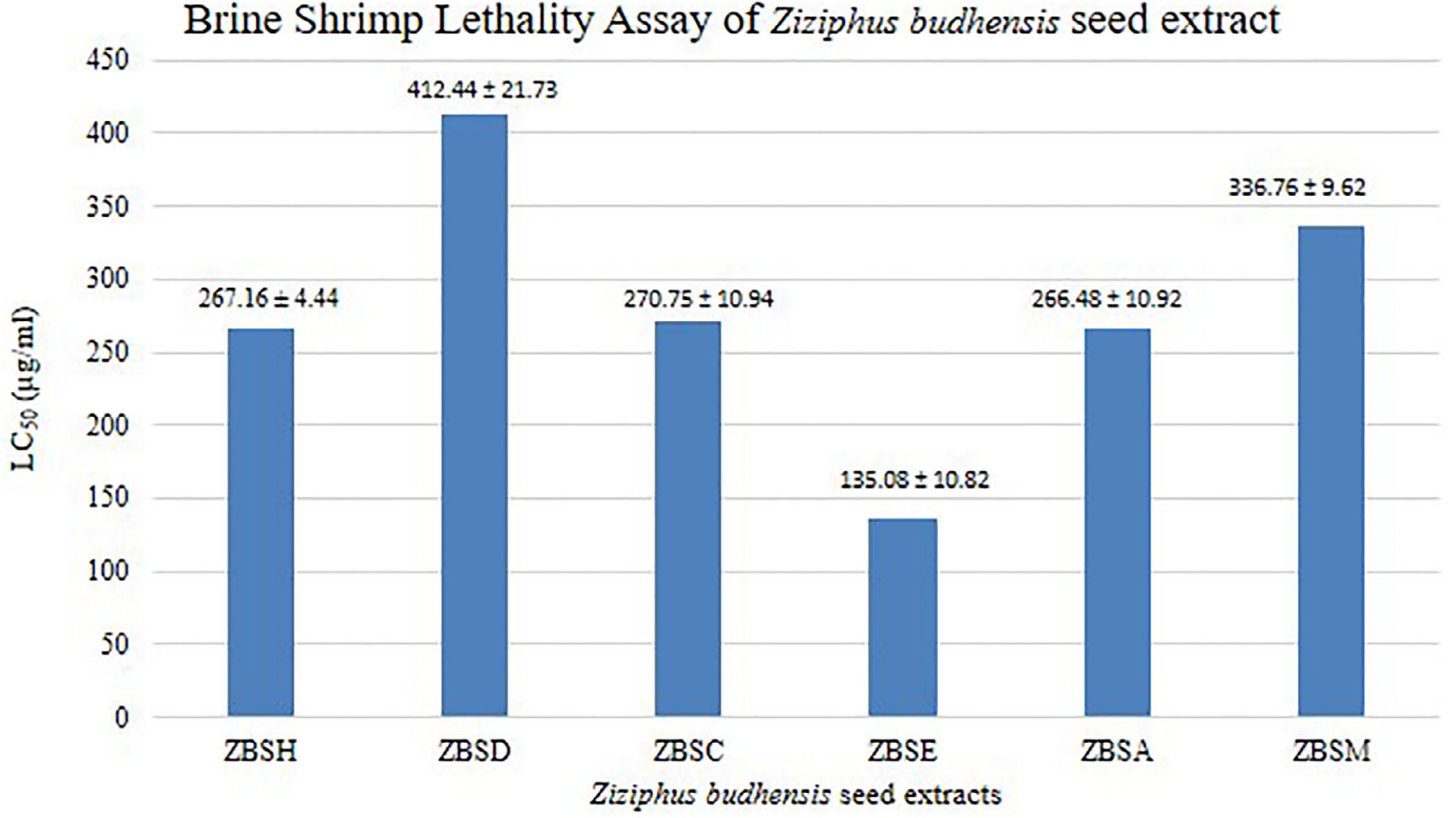
Brine shrimp lethality assay of the seed extracts of *Z. budhensis*. ZBSH: hexane, ZBSD: dichloromethane, ZBSC: chloroform, ZBSE: ethylacetate, ZBSA: acetone, ZBSM: methanol.

The Lethal concentration 50% (LC_50_) values for hexane, dichloromethane, chloroform, ethyl acetate, acetone, and methanol extracts of *Z. budhensis* seed were observed by exposing 10 freshly hatched nauplii to varying concentrations (62.5, 125, 250, 500, 1000 µg/mL) of each extract. The calculated LC_50_ values were 267.16 ± 4.44, 412.44 ± 21.73, 270.75 ± 10.94, 135.08 ± 10.82, 266.48 ± 10.92, and 336.76 ± 9.62 µg/mL, respectively and are shown in Figure 4.

These results demonstrate a dose concentration toxicity effect. A very low mortality effect was observed in nauplii while treating with the hexane, DCM, chloroform, and ethyl acetate extracts. In contrast, acetone and methanol showed no mortality at the same concentration. The hexane extract exhibited moderate toxicity, with an LC_50_ value of 267.16 ± 4.44 µg/mL, indicating a concentration‐dependent cytotoxic effect. The LC_50_ value of the DCM extract from the seed extract was found to be 412.44 ± 21.73 µg/mL, indicating the least toxicity among all extracts.

In the ethyl acetate extract, nauplii mortality began at 125 µg/mL and increased to 100% at the maximum concentration of 1000 µg/mL. The LC_50_ value of the ethyl acetate extract from the seed was 135.08 ± 10.82 µg/mL, indicating its toxicity. Ethyl acetate extract showed the highest toxicity among the extracts. This indicates that ethyl acetate extract was the most effective in causing mortality in brine shrimp at lower concentrations, suggesting it has the highest toxic potential among the extracts tested.

At a concentration of 1000 µg/mL, complete mortality was observed in all extracts except DCM. All extracts fall into the “Moderately Toxic” category (LC_50_ values between 100 and 500 µg/mL).

### 2.4 Antioxidant Screening Analysis

**FIGURE 5:**
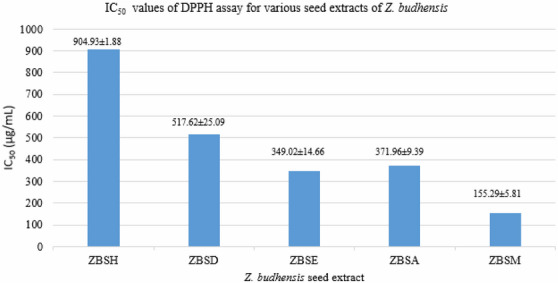
Antioxidant assay of Seed extracts of Z. budhensis. ZBSH: hexane, ZBSD: dichloromethane, ZBSC: chloroform, ZBSE: ethylacetate, ZBSA: acetone, ZBSM: methanol.

The in vitro antioxidant activity of each *Z. budhensis* seed extract is assessed by measuring IC_50_ using the DPPH method. The methanolic extract of *Z. budhensis* exhibited the most potent free radical scavenging activity, with an IC_50_ value of 155.29 ± 5.81 µg/mL, as shown in Table S4 and Figure 5.

Multiple research efforts have demonstrated a connection between the amount of phenolic compounds and antioxidant activity, highlighting the crucial function of these phenolics in plant defence mechanisms, especially through their role in limiting the buildup of reactive oxygen species (ROS) [16]. The different composition, as well as the different ability of the solvents to extract the phytoconstituents and mostly the polyphenolic constituents of the plant material, may explain the differences observed for the extract's activity.

Hexane extract (IC_50_ 904.93 ± 1.88 µg/mL) has weak antioxidant activity, and the dichloromethane extract (IC_50_ 517.62 ± 25.09 µg/mL) shows poor antioxidant activity as well. The ethyl acetate extract (IC_50_ 349.02 ± 14.66 µg/mL) and the acetone extract (IC_50_ 371.96 ± 9.39 µg/mL) exhibit moderate antioxidant activity.

Among all extracts, the methanol extract (155.29 ± 5.81 µg/mL) shows the strongest antioxidant activity, which can be partly explained by the presence of phenolic compounds and other phytoconstituents.

### 2.5 In Vitro Inhibition of *α‐*Amylase as a Preliminary Test to Evaluate Antidiabetic Compounds

In this study, an in vitro α‐amylase inhibitory assay was used to preliminarily evaluate the antidiabetic properties of seed extracts derived from Z. budhensis. The amylase‐inhibitory activity of each *Z. budhensis* seed extract is assessed by its IC_50_ value. The observed values are moderate; the chloroform extract did not show significant activity, while hexane at a higher tested concentration decreased enzyme activity by only 50%.

**FIGURE 6:**
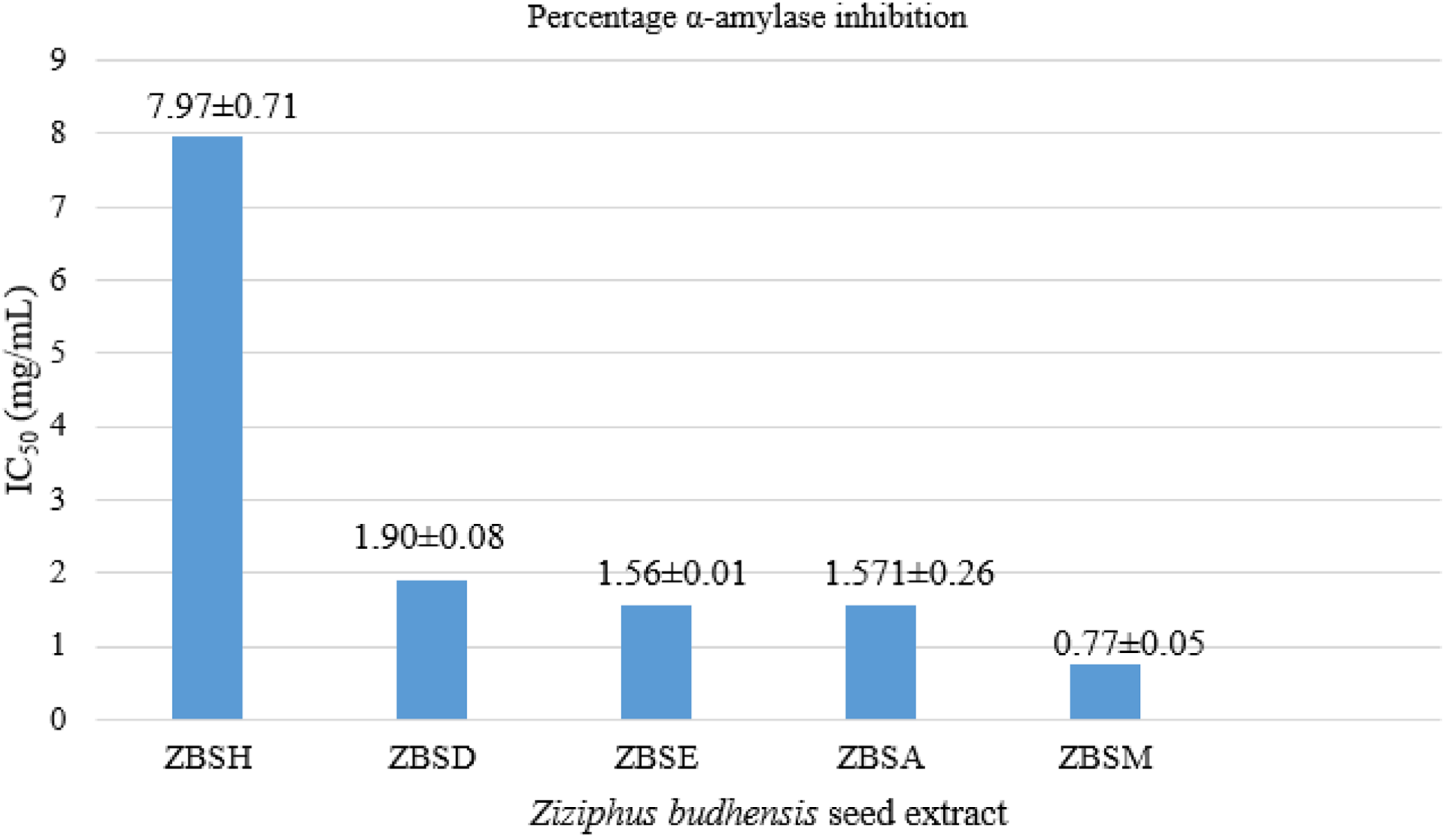
IC_50_ values for *α*‐amylase inhibition of different seed extracts of *Z. budhensis*. ZBSH: hexane, ZBSD: dichloromethane, ZBSC: chloroform, ZBSE: ethylacetate, ZBSA: acetone, ZBSM: methanol.

The hexane, dichloromethane, ethyl acetate, acetone, and methanol extracts of *Z. budhensis* were found to have IC_50_ values of 7.97 ± 0.71, 1.90 ± 0.08, 1.56 ± 0.01, 1.571 ± 0.26, and 0.77 ± 0.05 mg/mL, respectively (Table S5 and Figure 6). Chloroform extract shows no activity at the listed concentrations.

Hexane extract (IC_50_ 7.97 ± 0.71 mg/mL) exhibits the weakest amylase inhibitory activity, suggesting a low potential for antidiabetic effects. Dichloromethane extract (IC_50_ 1.90 ± 0.08 mg/mL) demonstrates moderate inhibitory properties, performing better than the hexane extract but not as well as ethyl acetate, acetone, or methanol.

Ethyl acetate extract (IC_50_ 1.57 ± 0.26 mg/mL) and acetone extract (IC_50_ 1.56 ± 0.01 mg/mL) both present moderate inhibitory activity. Among the tested extracts, methanol (IC_50_ = 0.77 ± 0.05 mg/mL) has the most significant antidiabetic potential.

Hence, from these preliminary data, the methanol extract can be considered the most promising for its antidiabetic potential and may contain some constituents acting in this regard. Further research could focus on separating and characterizing the active compounds in the methanol extract to explore their possible use in diabetes management (Table 2).

### 2.6 In Vivo Acute Oral Toxicity Study

**TABLE 2:** Median lethal dose (LD_50_) of different seed extracts of *Z. budhensis*. ZBSH: hexane, ZBSD: dichloromethane, ZBSC: chloroform, ZBSE: ethylacetate, ZBSA: acetone, ZBSM: methanol.

Extracts	LD_50_ (mg/kg BW)	Hazard Statement	Remarks
ZBSH	> 2000 mg/kg BW	If consumed, it might be harmful.	There were no deaths reported.
ZBSD	> 2000 mg/kg BW	If consumed, it might be harmful.	There were no deaths reported.
ZBSC	> 2000 mg/kg BW	If consumed, it might be harmful.	There were no deaths reported.
ZBSE	> 2000 mg/kg BW	If consumed, it might be harmful.	There were no deaths reported.
ZBSA	> 2000 mg/kg BW	If consumed, it might be harmful	There were no deaths reported.
ZBSM	> 2000 mg/kg BW	If consumed, it might be harmful	There were no deaths reported.

During an in vivo acute oral toxicity test in mice, no toxicity was observed with any of the six extracts at a dose of 2000 mg/kg.

## 3 Phytochemical Composition Studied by Liquid Chromatography Mass Spectrometry (LC‐MS)

**FIGURE 7:**
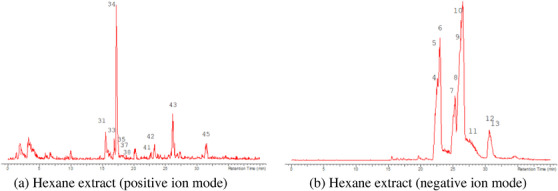
*Ziziphus budhensis* seeds hexane extract. Main peaks detected in (a) positive ion mode and (b) negative ion mode, numbers are referred to Table 3.

**FIGURE 8:**
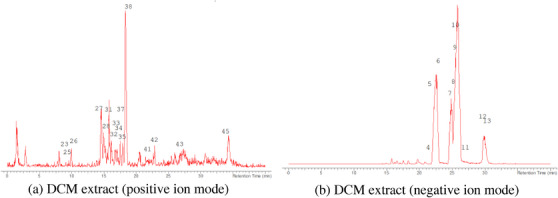
*Ziziphus budhensis* seeds DCM extract. Main peaks detected in (a) positive ion mode and (b) negative ion mode, numbers are referred to Table 3.

**FIGURE 9:**
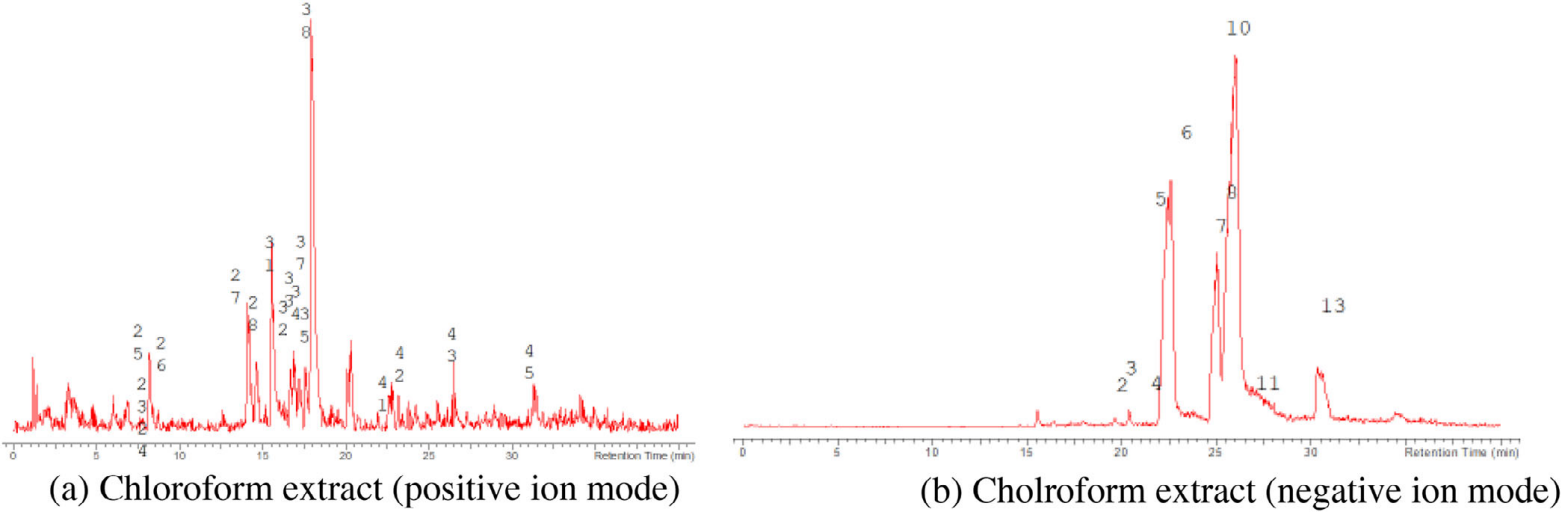
*Ziziphus budhensis* seeds chloroform extract. Main peaks detected in (a) positive ion mode and (b) negative ion mode, numbers are referred to Table 3.

**FIGURE 10:**
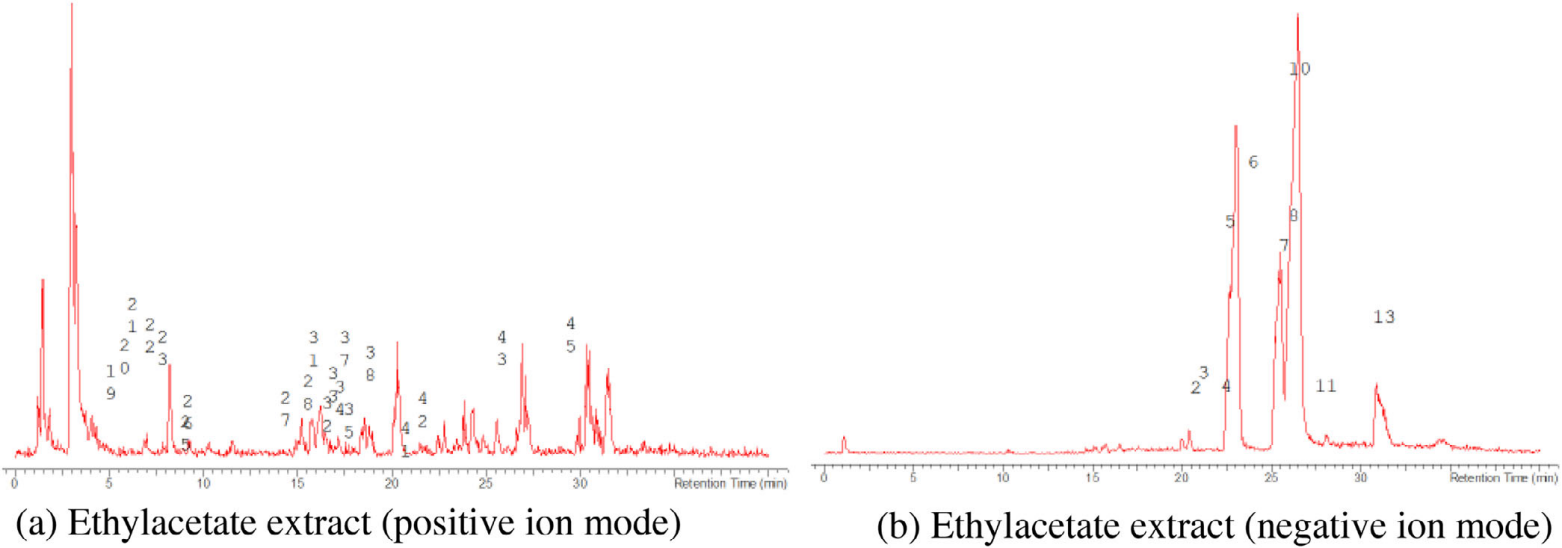
*Ziziphus budhensis* seeds ethylacetate extract. Main peaks detected in (a) positive ion mode and (b) negative ion mode, numbers are referred to Table 3.

**FIGURE 11:**
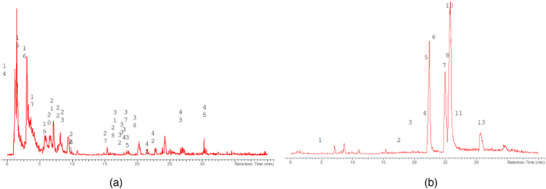
*Ziziphus budhensis* seeds acetone extract. Main peaks detected in (a) positive ion mode and (b) negative ion mode, numbers are referred to Table 3.

**FIGURE 12:**
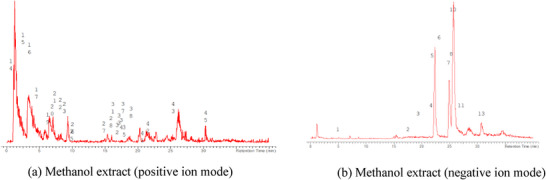
*Ziziphus budhensis* seeds methanol extract. Main peaks detected in (a) positive ion mode and (b) negative ion mode, numbers are referred to Table 3.

**FIGURE 13:**
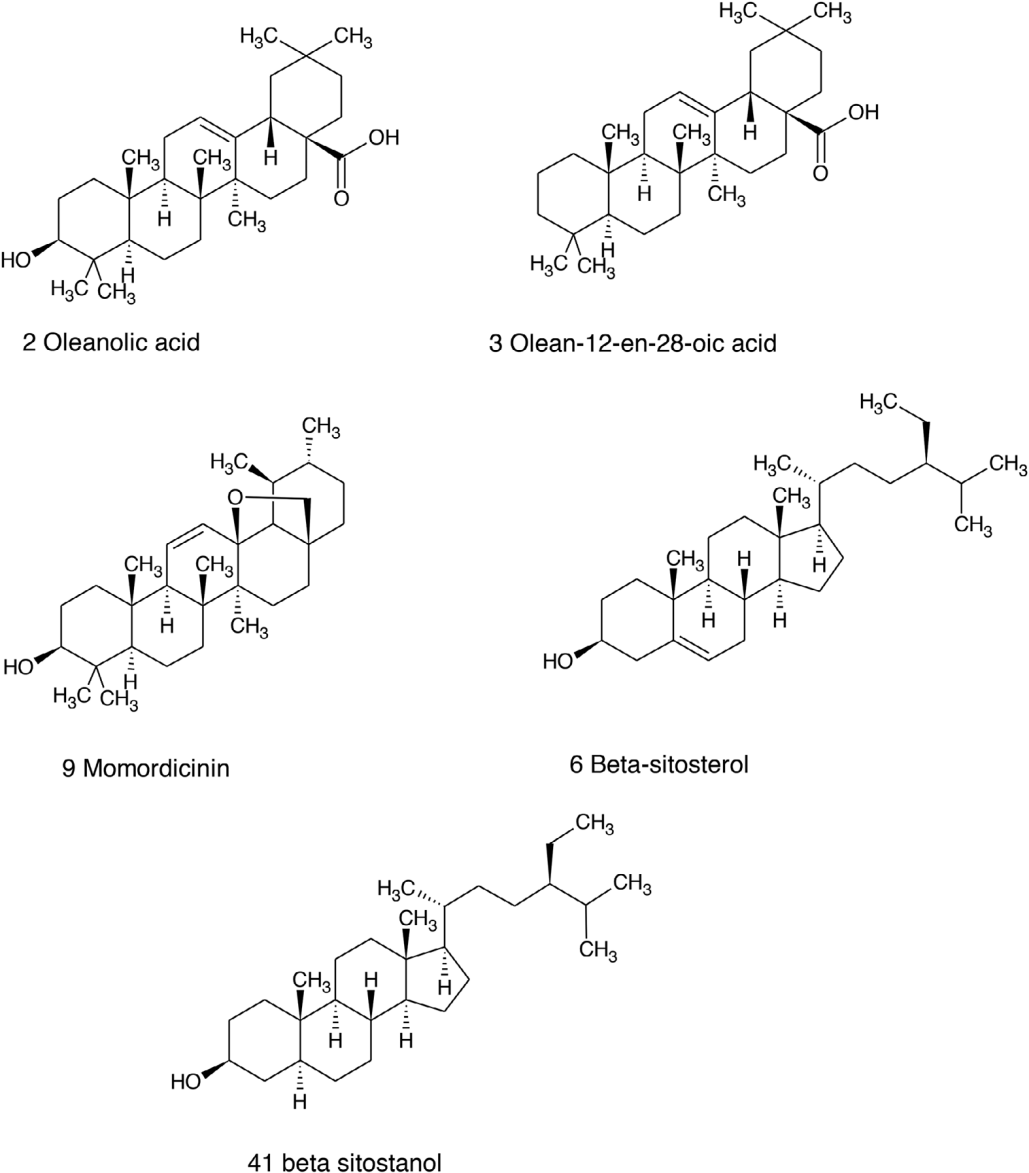
Structure of the triterpene and phytosterol derivatives detected in *Ziziphus* seeds.

**FIGURE 14:**
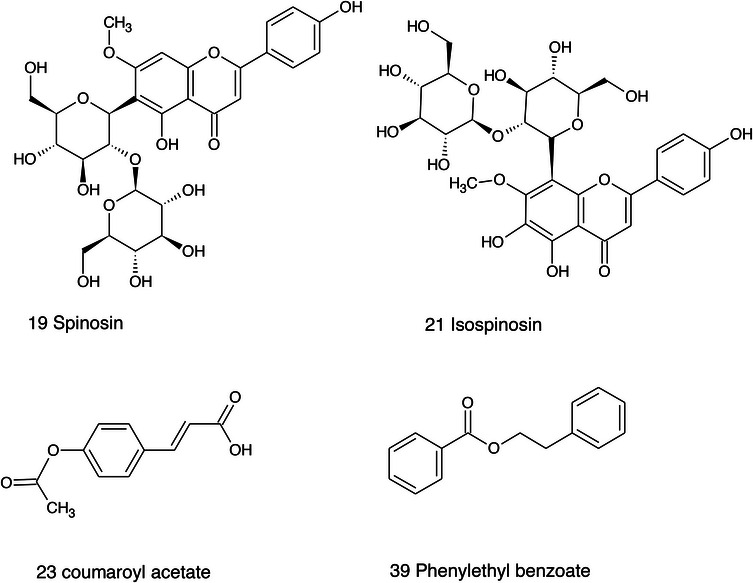
Structure of the glycosidic flavonoids and benzoic acid derivatives detected in *Ziziphus* seeds.

**FIGURE 15:**
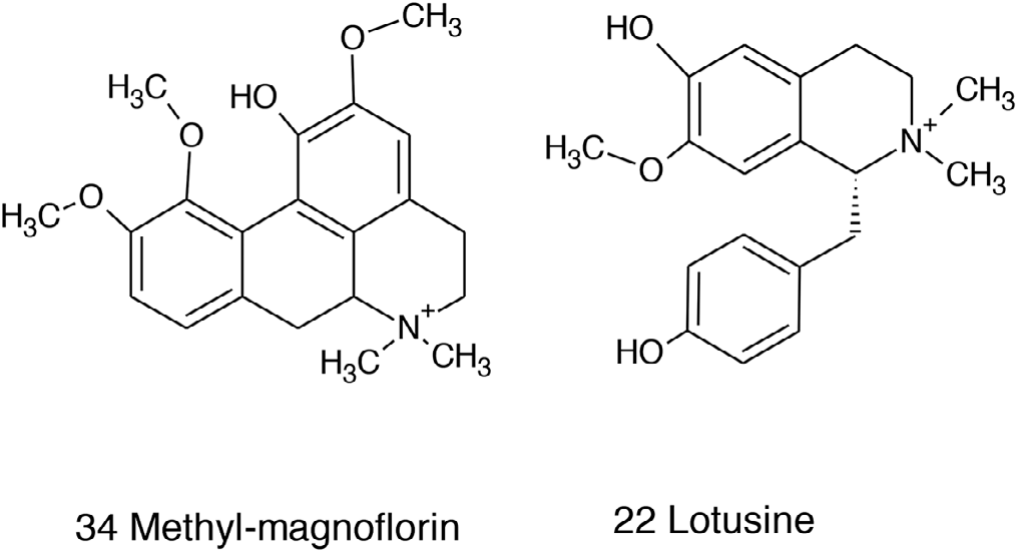
Structure of the cyclopeptide alkaloids and aporphyne alkaloid derivative detected in *Ziziphus* seeds.

The LC‐MS analysis of the seed extracts shows mostly the presence of fatty acid derivatives, as expected in seeds. Moreover, as demonstrated by the LC chromatograms, lipids are detectable in all extracts obtained with the different solvents. As reported in Figures 7, 8, 9, 10, 11, 12, 13, 14, 15, for all the extracts, a similar qualitative chromatographic profile is observed. Some compounds can be preferentially detected in extracts obtained with the more hydrophilic solvents. For example, the amino acid derivatives *N,N*‐dimethyl‐β‐alanine, arginylglycine, prolinetyrosine, and lysyltyrosine have been detected in the acetone and methanol extractions. The results indicate that the high lipid content makes the extracts obtained from the seeds a consistent source of these constituents.

Among the secondary metabolites, several triterpenoid derivatives are present, namely olean‐12‐en‐28‐oic acid, oleanolic acid, and momordicinin, which feature a further heterocyclic five‐membered oxygen‐bearing cycle. Also, as expected, phytosterols have been detected, namely beta‐sitosterol and sitostanol. All results are summarized in Table 3, where compounds are listed with numbers; the same numbers are highlighted in the following figures, which represent the chromatograms for each extract.

**TABLE 3:** Compounds observed in LC –MS of seed extracts of *Z. budhensis*, letters (x) in the extract column indicate the presence in the various solvents. (H: hexane, D: dichloromethane, C: chloroform, E: ethylacetate, A: acetone, M: methanol).

S.N	Rt	[M‐H]^−^	Molecular formula	Compounds	ZBSH	ZBSD	ZBSC	ZBSE	ZBSA	ZBSM
1.	3.27	295.0823	C_14_H_16_O_7_	*p*‐coumaroyl pentose					x	x
2.	22.66	455.3527	C_30_H_48_O_3_	Oleanolic acid			x	x	x	x
3.	22.86	439.3582	C_30_H_48_O_2_	Olean‐12‐en‐28‐oic acid			x	x	x	x
4.	23.1	279.2324	C_18_H_32_O_2_	Linoleic acid	x	x	x	x	x	X
5.	23.2	563.4681	C_35_H_64_O_5_	Diglyceride	x	x	x	x	x	X
6.	23.75	413.3789	C_29_H_50_O	Beta‐sitosterol	x	x	x	x	X	X
7.	25	255.234	C_16_H_32_O_2_	Palmitic acid	x	x	x	x	x	x
8.	25.3	281.2481	C_18_H_34_O_2_	Oleic acid	x	x	x	x	x	x
9.	25.37	437.3514	C_30_H_46_O_2_	Momordicinin (13,28‐Epoxyurs‐11‐en‐3‐one)	x	x	x	x	x	x
10.	26.3	483.40208	C_29_H_56_O_5_	Diglyceride derivative	x	x	x	x	x	x
11.	28.6	497.4475	C_65_H_120_O_6_	TG(22:1(13Z)/22:1(13Z)/18:1(11Z))	x	x	x	x	x	x
12.	30.2	283.2635	C_18_H_36_O_2_	Stearic acid	x	x	x	x	x	x
13.	30.5	[M‐3H]^−3^ 309,293	C_61_H_118_O_5_	Triglyceride	x	x	x	x	x	x
		**[M+H]^+^**			**ZBSH**	**ZBSD**	**ZBSC**	**ZBSE**	**ZBSA**	**ZBSM**
14.	1.7	117.0803	C_5_H_10_NO_2_	*N,N*‐dimethyl‐β‐alanine					x	x
15.	1.93	254.12240	C_8_H_17_N_5_O_3_	Arginylglycine					x	x
16.	2.92	[M+NH_4_‐H2O]^+^ 159,1123	C_7_H_13_NO_3_	Valerylglycine/Isovaleryl glycine					x	x
17.	3.27	342.20761	C_15_H_23_N_3_O_4_	Lysyltyrosine					x	x
18.	5.95	314.269	C_18_H_35_NO_3_	Palmitoylglycine					x	x
19.	7.08	609.3181	C_28_H_32_O_15_	Spinosin				x	x	x
20.	7.15	609.1967	C_32_H_32_O_12_	4‐(4‐Hydroxyphenyl)‐2‐butanone O‐[2‐galloyl‐6‐cinnamoylglucoside]				x	x	x
21.	7.65	609.3181	C_28_H_32_O_15_	Isospinosin				x	x	x
22.	8.11	314.25443	C_19_H_24_NO_3_	Lotusine				x	x	
23.	8.14	193.0859	C_11_H_12_O_3_	coumaryl acetate			x	x	x	x
24.	8.14	709.39327	C_38_H_60_O_12_	triterpenoid saponin				x	x	
25.	9.4	448.2733	C_26_H_39_O_6_	16‐feruloyloxypalmitate			x	x	x	
26.	9.86	197.0444	C_9_H_8_O_5_	Hydroxycaffeic acid			x	x	x	x
27.	14.5	558.3863	C_29_H_43_N_5_O_6_	nummularine‐P (Ib)		x		x	x	x
28.	14.9	592.3842	C_32_H_41_N_5_O_6_	nummularine‐B		x		x	x	x
29.	15.5	441.6623	C_29_H_60_O_2_	Nonacosanediol		x	x	x		
30.	15.8	279.3182	C_20_H_38_	Neophytadiene		x	x			
31.	15.8	445.3312	C_28_H_44_O_4_	5,6:8,9‐Diepoxyergost‐22‐ene‐3,7alpha‐diol	x	x	x	x	x	
32.	16.1	501.1402	C_25_H_26_O_11_	P‐Coumaric acid‐O‐glucoside‐glucuronide					x	x
33.	16.9	297.2424	C_18_H_32_O_3_	Hydroxyoctadecadienoic acid	x	x	x	x	x	
34.	17.31	358.2013	C_21_H_27_NO_4_	methyl‐magnoflorine	x	x	x	x	x	
35.	17.8	[M+H+2Na]^2+^ 297.3502	C_54_H_100_O_6_	TG(15:0/18:1(9Z)/18:1(9Z))[iso3]	x			x	x	
36.	18.2	305.3481	C_16_H_20_N_2_O	Peptide derivative (Pro‐Try)				x	x	x
37.	18.2	628.4935	C_37_H_62_O_5_	DG(16:1n7/0:0/18:4n3)	x	x	x			
38.	18.22	[M+H‐H2O]^+^ 628,4706	C_35_H_68_NO_7_P	Phosphatidyl ethanolamine derivative	x	x	x	x	x	x
39.	18.3	305.1209	C_15_H_14_O_2_	phenylethylbenzoate		x	x	x	x	x
40.	18.6	281.3201	C_20_H_40_	10‐Eicosene	x	x	x	x		
41.	22.8	439.3928	C_29_H_52_ONa	Beta‐sitostanol	x	x	x	x	x	x
42.	23.7	355.2741	C_21_H_38_O_4_	MG(18:2(9Z,12Z)/0:0/0:0)	x	x	x	x	x	x
43.	26.8	469.4582	C_31_H_58_O_2_	(Z)‐22‐Hentriacontene‐2,4‐dione	x	x	x	x	x	x
44.	32.6	329.2334	C_18_H_34_O_5_	(15Z)‐9,12,13‐trihydroxy‐15‐ octadecenoic acid		x	x	x	x	x
45.	34.3	391.3183	C_46_H_88_O6	TG(i‐19:0/i‐16:0/8:0)	x	x	x	x	x	x

The most significant triterpenoids and phytosterol structures are reported in Figure 13.

The extracts obtained with more hydrophilic solvents allow the detection of two C‐glycosidic flavonoids, namely spinosin and isospinosin, which are known to be present in *Ziziphus*. In the same extracts, some hydroxycinnamic acid or benzoic acid derivatives have been detected, namely *p*‐coumaric acid‐*O*‐glucoside‐glucuronide, *p*‐coumaroyl acetate, and phenylethylbenzoate (Figure 14).

A limited number of alkaloid derivatives have also been detected, namely the cyclopeptide alkaloids nummularins, detected in extracts obtained with dichloromethane, acetone, and methanol; the aporphine alkaloid derivative methyl‐magnoflorine; and the isoquinoline lotusine.

## 4 Discussion

To our knowledge, this is the first study to comprehensively characterize the phytochemistry and biological potential of *Z. budhensis* seeds. The results for the different extracts from *Ziziphus* seeds showed limited responses in the tested bioactivities. The most promising effect appears to be the antibacterial and antifungal activity observed in the acetone extract. Limited inhibitory activity is observed against the amylase enzyme, and limited antioxidant effects are registered in the DPPH assay. In this regard, the most significant results have been obtained from the methanol extract, but the observed bioactivity is relatively low. From a phytochemical point of view, the high lipid content in the seed tissue negatively influences the extraction of other constituents, which are present in limited amounts compared to lipids. Sequential extraction with solvents of increasing polarity enabled the extraction of different classes of compounds, including lipophilic and hydrophilic constituents. Nevertheless, an apparent prevalence of fatty acids, particularly linoleic (n‐6) and oleic (n‐9) acids, is evident in all the obtained extracts, indicating that seeds can be considered a significant source of these lipids. LC‐MS analysis, in any case, allowed the detection of peptides, triterpenoids, phenolic compounds, cyclopeptide alkaloids, and phytosterols.

Related to the most significantly observed activities, the antibacterial and antifungal effects recorded for the acetone and ethyl acetate extracts were considered based on the obtained composition analysis. As shown in Table 3, some compounds, mainly detected in the acetone, ethyl acetate, and methanol extracts, have previously been reported to have antibacterial activity.

Direct comparisons with previously published studies on *Z. budhensis* are limited due to the scarcity of phytochemical and pharmacological investigations on this species. Most earlier studies focused on its taxonomy, ecology, seed germination, and socioeconomic importance rather than medicinal properties [9, 10, 11, 12]. Consequently, the present work represents the first systematic evaluation of the phytochemical composition and biological activities of *Z. budhensis* seeds.

Our findings complement and extend earlier results from *Z. budhensis* leaves, which showed greater phenolic diversity and more potent antioxidant activity [13]. In contrast, the seed extracts analyzed in the present study were dominated by fatty acids and lipophilic constituents, with relatively limited antioxidant and enzyme‐inhibitory activities but notable antibacterial and antifungal effects, particularly in acetone and ethyl acetate extracts. These observations indicate an apparent plant‐part‐dependent variation in chemical composition and bioactivity within *Z. budhensis*, consistent with trends reported in other members of the genus.

In the absence of extensive prior studies on *Z. budhensis* seeds, comparisons with other *Ziziphus* species are both necessary and appropriate. The detection of cyclopeptide alkaloids, triterpenoids, phytosterols, and flavonoids in the seed extracts aligns with reports from related species such as *Z. mauritiana, Z. jujuba*, and *Z. spina‐christi*, in which these compounds have been associated with antimicrobial and antioxidant activities [8, 17, 18]. However, the present study uniquely demonstrates that *Z. budhensis* seeds, despite their primary cultural use, also represent a potential source of bioactive metabolites with antimicrobial relevance. The flavonoids spinosin and isospinosin may, at least in part, contribute to the observed antibacterial activity, as previous studies have demonstrated the antibacterial roles and underlying mechanisms of action of flavonoids [19]. Moreover, the coexistence of antioxidant and other biological activities, together with the presence of unsaturated lipids, suggests that *Ziziphus* seeds may also hold potential for skin‐related or cosmetic applications. Similar multifunctional properties have been reported for *Z. jujuba*, supporting the broader applicability of bioactive seed constituents within the genus [20].

The identification of specific bioactive constituents, such as cyclopeptide alkaloids and flavonoids, provides a basis for future targeted studies aimed at isolating and characterizing individual compounds responsible for the observed antimicrobial effects. Furthermore, given the traditional cultural and economic importance of *Z. budhensis* in Nepal, the development of seed‐derived products could add value to local communities while supporting conservation of this endemic species.

## 5 Conclusion

*Ziziphus budhensis* is an important plant for the Nepal territory, having symbolic spiritual and religious values, as well as being significant from an economic point of view. The seeds are edible and used for religious purposes, but they can also be considered a source of bioactive compounds. The results show that the seeds are chemically dominated by unsaturated fatty acids, namely the n‐6 linoleic and the n‐9 oleic acids. In addition, several classes of secondary metabolites are detected in the more lipophilic extracts, namely phytosterols and triterpenoids, which are commonly associated with biological activity. Furthermore, using extraction solvents with high polarity can also extract peptides and phenolic compounds. The preliminary bioassays, shown as relevant results, indicate no toxicity in the different tests performed, suggesting a possible high safety profile for this vegetal material. Although the antioxidant and α‐amylase inhibitory responses were not particularly strong, certain extracts demonstrated apparent antibacterial and antifungal effects, suggesting selective bioactivity rather than broad‐spectrum efficacy. The chemical analysis and observed bioactivities indicate the need for future studies on the application of *Ziziphus* seeds, especially as an antibacterial agent and as a vegetal source of bioactive compounds for cosmetic and skin applications. Overall, the seeds of *Z. budhensis* hold potential not only as antimicrobial agents but also as a sustainable natural resource for pharmaceutical and cosmetic industries, contributing both scientific and socioeconomic value to Nepal.

Seeds were dominated by unsaturated fatty acids with moderate antimicrobial activity, while leaves showed greater chemical diversity, including quercetin derivatives and alkaloids, with more potent antioxidant and antimicrobial effects. Both plant parts demonstrated moderate cytotoxicity in brine shrimp but were safe in vivo up to 2000 mg/kg. Overall, *Z. budhensis* represents a multifunctional source of bioactive metabolites with promising nutraceutical, pharmaceutical, and cosmetic potential, warranting further studies on compound isolation and formulation development. Future studies focusing on compound isolation, mechanism of action, and formulation development would be essential to explore further and substantiate these applications, while also supporting the sustainable utilization of this culturally significant Himalayan species.

## 6 Experimental Section

### 6.1 Collection of the Plant Materials

Seeds of *Z. budhensis* (Rhamnaceae) were placed together from Timal, Kavrepalanchowk District, Nepal (1000–2000 m; 27°32’44.52'' N, 85°38’00.96'' E) in March 2021. The plant materials were authenticated by Ms. Rita Chhetri, Research Officer, the National Herbarium & Plant Laboratories (KATH), Nepal, on August 08, 2024. A voucher specimen no. S‐202 has been deposited at KATH [13].

### 6.2 Materials and Extraction Techniques

The seeds were initially separated from the pericarp by breaking the endocarp and isolating the inner kernels. Collected seeds were shade‐dried at normal temperature (25°C–30°C) for 14 days and subsequently ground into an adequate powder using a grinding machine. The percentage yield of each extract was calculated on a dry weight basis using the formula:

$$\%\;\mathrm{yield}=\;\;\frac{dry\;weight\;of\;extract}{dry\;weight\;of\;plant}\;\;\times \;100\%$$

**FIGURE 16:**
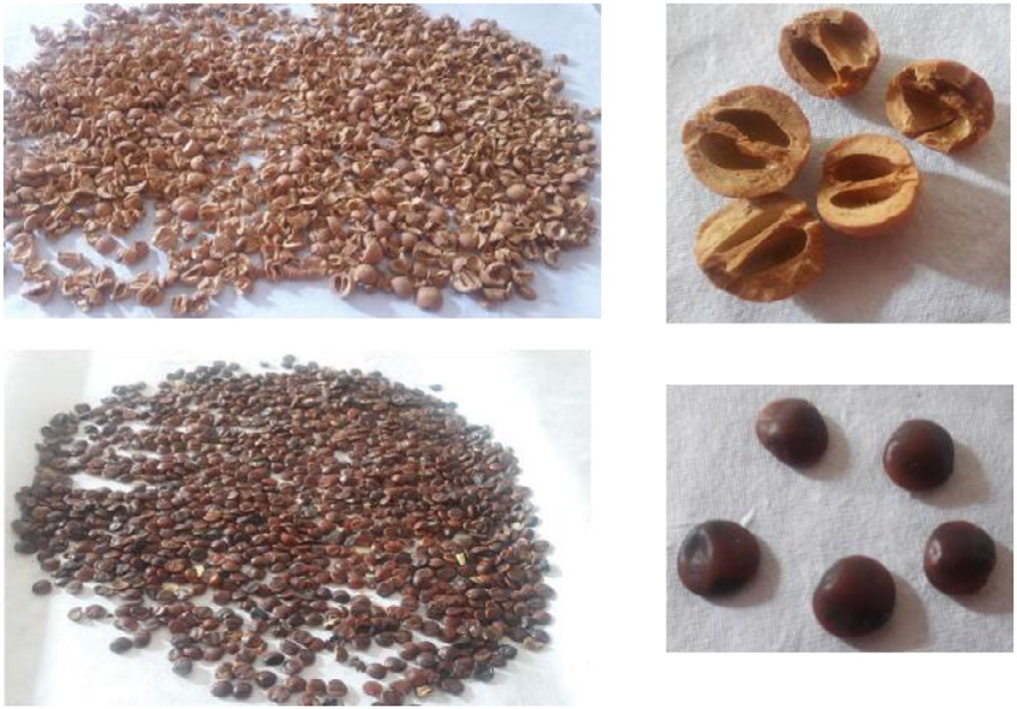
Seed part of *Z. budhensis*.

A total of 1.5 kg of powdered seed material was transferred into a 5000 mL conical flask and subjected to ultrasonic extraction with 3.5 L of hexane through an ultrasonic bath (40 kHz). The solvent was separated by a filtration process and concentrated under reduced pressure using a rotary evaporator. The residual seed (marc) was then successively extracted with dichloromethane, chloroform, ethyl acetate, acetone, and methanol, following the same procedure. In this way, six crude extracts were obtained in order of increasing polarity, namely hexane (ZBSH), dichloromethane (ZBSD), chloroform (ZBSC), ethyl acetate (ZBSE), acetone (ZBSA), and methanol (ZBSM). All extracts were concentrated to dryness and stored at room temperature for subsequent analyses (Figure 16).

### 6.3 Total Phenolic Content

Total phenolic content in all six *Z. budhensis* extracts was examined using the Folin–Ciocalteu assay. A stock solution of gallic acid (1 mg/mL) was prepared by dissolving 1 mg in 1 mL of distilled water. Then, 1 mL of this standard in methanol was combined with 1 mL of Folin–Ciocalteu reagent (previously diluted 1:10 with distilled water) and 0.8 mL of 1 M sodium carbonate (Na_2_CO_3_). The reaction mixture was incubated in the dark for 15 min, and absorbance was recorded at 765 nm. Results were stated as gallic acid equivalents per gram of dry extract [15, 21, 22, 23].

By following this method, the total phenolic content was measured in milligrams of gallic acid equivalent in all samples:

$$\mathrm{TPC}=\frac{c\times v}{m}$$

where *c* = concentration of gallic acid from the curve (mg/mL), *v* = volume of extract (mL), *m* = weight of plant extract (g)

### 6.4 Total Flavonoid Content

The total flavonoid content of the *Z. budhensis* extracts was quantified using an aluminium chloride (AlCl_3_) colorimetric assay. Each extract (0.1 mg/mL in methanol, 1 mL) was combined with 1 mL of 10% AlCl_3_, and the mixture was permitted to react for 1 h at the standard temperature. Absorbance at 420 nm was measured using a methanol blank as a control. Quercetin was used as the calibration standard, and results were reported as mg quercetin equivalents per gram of solid extract [24, 25].

The total flavonoid content was determined using this calculation:

$$\mathrm{TFC}=\frac{c\times v}{m}$$

where *c* = concentration of quercetin from curve (mg/mL), *v* = volume of extract (mL), and *m* = weight of plant extract (g).

### 6.5 Antimicrobial Activity

Antimicrobial activity was evaluated using the agar well diffusion method.

### 6.6 Preparation of Microbial Culture Media

Liquid Broth (LB) was made ready by dissolving 13 g of LB powder (Sisco Research Laboratories Pvt. Ltd., India) in 1 L of refined water. The medium was sterilized by autoclaving at 121°C and 15 psi for 25 min. After cooling to 40°C–50°C, 5 mL aliquots were dispensed into sterile 15 mL Falcon tubes. These were inoculated with bacterial seed cultures and incubated for 24 h to obtain actively growing cultures [26].

### 6.7 Preparation of MH Media Plates and Antimicrobial Assay

Mueller–Hinton agar (MHA) plates were prepared by dissolving 39 g of MH agar powder (Sisco Research Laboratories Pvt. Ltd., India) in 1 L of distilled water, then sterilizing at 121°C and 15 psi for 25 min. The medium was chilled to 40°C–50°C, then poured into Petri dishes (25 mL per plate) and refrigerated until further use. For antimicrobial testing, 150 µL of bacterial seed culture was consistently distributed across each plate with a sterile cotton swab. Wells were prepared in the agar, and 100 µL of the extract samples were added to each well. Kanamycin (10 µL, 5 mg/mL) was placed as a positive control. The antibacterial activity of the plates was assessed by measuring the zones of inhibition after incubation for 24 h at 37°C [27].

### 6.8 Brine Shrimp Lethality Assay

The cytotoxic potential of the *Z. budhensis* extracts was calculated using the brine shrimp (*Artemia salina*) lethality bioassay. *Artemia salina* nauplii hatched in artificial seawater under constant aeration and illumination. Groups of 10 nauplii were exposed to different concentrations of each extract (62.5–1000 µg/mL). Brine shrimp nauplii were placed in different concentrations of the extracts for 24 h, after which mortality was assessed by counting the surviving (motile) nauplii. The median lethal concentration (LC_50_) was calculated using regression analysis, with values below 1000 µg/mL considered indicative of significant cytotoxicity [28, 29, 30, 31, 32, 33].

### 6.9 Antioxidant Activity

The in vitro antioxidant activity of *Z. budhensis* seed extracts was assessed using the 2,2‐diphenyl‐1‐picrylhydrazyl (DPPH) free radical scavenging assay. Ascorbic acid was used as the standard antioxidant reference compound and evaluated in parallel with the extracts.

Stock solutions of each *Z. budhensis* extract (1.5 mg/mL in methanol) were prepared, from which serial dilutions were made at concentrations of 1000, 500, 250, 125, 62.5, and 31.25 µg/mL. To perform the assay, 500 µL of each extract concentration was added to 1500 µL of 0.1 mM DPPH in methanol. The reaction mixtures were homogenized for 2 min before incubation and then protected from light with aluminium foil, and incubated at standard temperature for 30 min. Methanol was used as the blank when measuring absorbance at 517 nm using a UV–visible spectrophotometer. 1.5 mL of DPPH solution and 0.5 mL of methanol were combined to form a control, and the mixture's absorbance was measured.

The following formula was used to determine the percentage of radical scavenging activity:

$$\mathrm{Percentage}\;\mathrm{scavenging}=\frac{\left(A_{o}-A_{T}\right)}{A_{o}}\;\times \;100\%$$

where, A_0_ = Absorbance of the DPPH and A_T_ = absorbance of the DPPH free radical solution containing the sample extract.

The IC_50_ value (the concentration required to scavenge 50% of DPPH radicals) was determined by plotting concentration vs % scavenging and using a dose‐response curve [34, 35].

### 6.10 In Vitro Inhibition of *α*‐Amylase

The α‐amylase inhibitory activity of *Z. budhensis* seed extracts was evaluated using the 3,5‐dinitrosalicylic acid (DNSA) calorimetric method. Acarbose was employed as the standard reference inhibitor. The seed extracts (diluted in at least 10% Dimethyl sulfoxide, DMSO) were mixed with buffer and NaCl (pH ∼6.9) to provide a series of concentrations. A 200 µL portion of each concentration was pre‐incubated with the α‐amylase enzyme at 30°C for 10 min. Then, 200 µL of 1% (w/v) starch solution was kept and allowed to react for 3 min. The reaction was stopped by adding 200 µL of DNSA reagent, after which samples were heated in an 85°C–90°C water bath for 10 min. Once cooled to room temperature, each sample was diluted with 5 mL of distilled water. Absorbance was measured at 540 nm, with a blank containing buffer (instead of plant extract) defining 100% enzyme activity [36].

The percentage of α‐amylase inhibition was considered using the following equation:

$$\begin{array}{ccc} \%\;\alpha -\mathrm{amylase}\;\;\mathrm{inhibition} & = & \frac{(\mathrm{Abs}100\%\mathrm{control}\;-\;\mathrm{AbsSample})}{\left(\mathrm{Abs}100\%\mathrm{Control}\right)}\\ & & \times \;100\% \end{array}$$

The IC_50_ values, which indicate the concentration at which *α*‐amylase inhibition is 50%, were obtained by plotting extract concentration against the percentage of *α*‐amylase inhibition.

### 6.11 In Vivo Acute Oral Toxicity Study

Acute oral toxicity was measured using the OECD (Organisation for Economic Co‐operation and Development) acute toxic class method 425 in accordance with the Chemical Testing Guidelines. The study was carried out on mice at the Pharmacology Laboratory of the Natural Product Research Laboratory (NPRL), Thapathali, Nepal. The experiments were approved by the Department of Plant Resources, Natural Products Research, Government of Nepal, Ministry of Forest & Environment (177/080/81). Before the experiment, the animals were not fed for 12 h, and body weights were recorded immediately before extract administration. The mice were randomly assigned into two groups: a control group receiving physiological saline and an investigational group receiving the extract via an orogastric (OG) tube at a dose of 2000 mg/kg body weight.

All animals were examined clinically four times daily, with careful assessment of behaviour, general health, nasal mucosa, skin and fur condition, respiratory rate, and somatomotor activity. Additional clinical observations included tremors, convulsions, diarrhoea, fatigue, excessive salivation, reduced responsiveness to stimuli, altered sleep–wake patterns, photoreactivity, and coma. Additionally, an abdominal palpation was done. The experimental group received a second dose of the extract at 2000 mg/kg, followed by a 48 h observation period during which no adverse effects were observed. All of the animals were mercifully put to death at the conclusion of the study [37, 38, 39] (Table 4).

**TABLE 4:** Substance classification based on the globally harmonized system for classification and labelling of chemicals (GHS) third edition guidelines [37].

Ranges (mg/kg)	Category	Classification	Hazard statement
> 2000 mg/kg	Category 5	Unclassified	Potentially harmful if consumed

> 300 ≤ 2000 mg/kg	Category 4	Hazardous	May be harmful if swallowed
> 50 ≤ 300 mg/kg	Category 3	Toxic	Ingestion may result in toxicity

> 5 ≤ 50 mg/kg	Category 2	Extremely toxic	Could be fatal if consumed.
< 5 mg/kg	Category 1	Severely toxic	Could be fatal if consumed.

## 7 Liquid Chromatography Mass Chromatography (LCMS)

For the liquid chromatography mass spectrometry analysis, a Waters ACQUITY UPLC system was used, coupled with a quadrupole time of flight Xevo operating in electrospray mode, adapting methodologies used in previous work [13]. The separation was implemented because the seed extracts contained higher lipid levels than the leaves and other organs. To update the current method, we provide an accurate description of the proposed methodology. As a stationary phase, an Accucore C18 column (150 × 2.1 mm, 2.6 µm) was used, and the column oven was set to 40°C. As mobile phases, water, 0.1% formic acid, and methanol were used, with a gradient from 90% water to 90% methanol over 25 min. Flow rate was 0.1 mL/min. As reference compounds previously studied, constituents obtained from *Ziziphus* species were used, and standard compounds available in the laboratory, such as spinosin, oleanolic acid, beta‐sitosterol, and beta‐sitostanol, were used to confirm the identification of the compounds [13]. Identified and confirmed compounds with references are listed in Table 3.

## Author Contributions

**Samjhana Bharati**: conceptualization, lab work, manuscript preparation, review and editing. **Binita Maharjan**: manuscript preparation, review and editing. **Timila Shrestha**: lab work. **Gopal Gautam Khatri**: lab work. **Hari Prasad Devkota**: review. **Ram Lal Swagat Shrestha**: conceptualization, review and editing, manuscript preparation, supervision. **Stefano Dall'Acqua**: conceptualization, manuscript preparation, supervision, review and editing

## Conflicts of Interest

The authors declare no conflicts of interest.

## Data Availability

Upon reasonable request, the corresponding author will provide the data supporting the study's conclusions.

## References

[1] M. S. Butler , “The Role of Natural Product Chemistry in Drug Discovery †,” Journal of Natural Products 67 (2004): 2141–2153. 15620274 10.1021/np040106y

[2] D. J. Newman and G. M. Cragg , “Natural Products as Sources of New Drugs Over the Nearly Four Decades From 01,” Journal of Natural Products 83 (2020): 770–803. 32162523 10.1021/acs.jnatprod.9b01285

[3] G. M. Cragg and D. J. Newman , “Biodiversity: A Continuing Source of Novel Drug Leads,” Pure and Applied Chemistry 77 (2005): 7–24.

[4] D. J. Newman and G. M. Cragg , “Natural Products as Sources of New Drugs From 1981 to 2014,” Journal of Natural Products 79 (2016): 629–661. 26852623 10.1021/acs.jnatprod.5b01055

[5] S. T. Sakna , A. Mocan , H. N. Sultani , N. M. El‐fiky , L. A. Wessjohann , and M. A. Farag , “Metabolites Profiling of Ziziphus Leaf Taxa via UHPLC/PDA/ESI‐MS in Relation to Their Biological Activities,” Food Chemistry 293 (2019): 233–246. 31151607 10.1016/j.foodchem.2019.04.097

[6] A. Alsayari and S. Wahab , “Genus Ziziphus for the Treatment of Chronic Inflammatory Diseases,” Saudi Journal of Biological Science 28 (2021): 6897–6914. 10.1016/j.sjbs.2021.07.076 PMC8626254 34866990

[7] B. Aggarwal , P. Sharma , and H. Lamba , “Ethanobotanical, Phytochemical and Pharmacological Properties of Zizyphus Nummularia (Burm. F.): A Review,” International Journal of Phytomedicine 10 (2018): 137–147.

[8] S. T. Sakna , Y. R. Maghraby , M. S. Abdelfattah , and M. A. Farag , “Phytochemical Diversity and Pharmacological Effects of Triterpenes From Genus Ziziphus: A Comprehensive Review,” Phytochemistry Reviews 22 (2023): 1611–1636.

[9] K. R. Bhattarai and M. L. Pathak , “A New Species of Ziziphus (Rhamnaceae) From Nepal Himalayas,” Indian Journal of Plant Sciences 4 (2015): 71–77.

[10] F. Lama , M. Koirala , M. L. Pathak , K. K. Pokharel , and S. Chaudhary , “Status and Ecological Preferences of “Bodhichitta” (Ziziphus species): A Case Study From Pokhari Narayansthan VDC, Timal, Kavrepalanchowk, Central Nepal,” Indian Journal of Plant Sciences 8 (2019): 12–19.

[11] H. Neupane , K. S. Thing , N. Upadhayay , and J. J. Gairhe , “Status of Bodichitta (Ziziphus budhensis) Cultivation and Its Prospects in Nepal,” Asian Journal of Agricultural Extension, Economics and Sociology 29 (2019): 1–11.

[12] T. Dorji , M. K. Lama , and N. Dorji , “Breaking the Stone : Overcoming Seed Dormancy and Seedling Emergence of the Rare Ziziphus Budhensis in Bhutan Himalayas,” Indian Journal of Plant Sciences 4 (2015): 47–54.

[13] S. Bharati , B. Maharjan , T. Shrestha , et al., “LC‐DAD‐MSn and HR‐LC‐QTOF Analysis of Ziziphus Budhensis Leaves and Evaluation of Their In Vitro and In Vivo Biological Activities,” Chemistry and Biodiversity 22 (2025): e202402835, 10.1002/cbdv.202402835. 40193212 PMC12351441

[14] K. H. Miean and S. Mohamed , “Flavonoid (myricetin, quercetin, kaempferol, luteolin, and apigenin) Content of Edible Tropical Plants,” Journal of Agricultural and Food Chemistry 49 (2001): 3106–3112, 10.1021/jf000892m. 11410016

[15] S. King , H. Rob , S. Lallianrawna , et al., “Determination of Total Phenolic Content, Total Flavonoid Content and Total Antioxidant Capacity of Ageratina Adenophora,” Science Vision 13 (2013): 149–156.

[16] M. Sengul , H. Yildiz , N. Gungor , B. Cetin , Z. Eser , and S. Ercisli , “Total Phenolic Content, Antioxidant and Antimicrobial Activities of Some Medicinal Plants,” Pakistan Journal of Pharmaceutical Sciences 22 (2009): 102–106. 19168430

[17] P. Panseeta , K. Lomchoey , S. Prabpai , et al., “Antiplasmodial and Antimycobacterial Cyclopeptide Alkaloids From the Root of *Ziziphus mauritiana* ,” Phytochemistry 72 (2011): 909–915. 21450320 10.1016/j.phytochem.2011.03.003

[18] A. Rajaei , D. Salarbashi , N. Asrari , B. S. Fazly Bazzaz , S. M. Aboutorabzade , and R. Shaddel , “Antioxidant, Antimicrobial, and Cytotoxic Activities of Extracts From the Seed and Pulp of Jujube (Ziziphus jujuba) Grown in Iran,” Food Science and Nutrition 9 (2021): 682–691. 33598153 10.1002/fsn3.2031 PMC7866595

[19] Y. Yan , X. Xia , A. Fatima , et al., “Antibacterial Activity and Mechanisms of Plant Flavonoids Against Gram‐Negative Bacteria Based on the Antibacterial Statistical Model,” Pharmaceuticals 17 (2024): 292, 10.3390/ph17030292. 38543078 PMC10974178

[20] D. Batovska , A. Gerasimova , and K. Nikolova , “Exploring the Therapeutic Potential of Jujube (Ziziphus jujuba Mill.) Extracts in Cosmetics: A Review of Bioactive Properties for Skin and Hair Wellness,” Cosmetics 11 (2024): 181, 10.3390/cosmetics11050181.

[21] V. Banothu , C. Neelagiri , U. Adepally , J. Lingam , and K. Bommareddy , “Phytochemical Screening and Evaluation of In Vitro Antioxidant and Antimicrobial Activities of the Indigenous Medicinal Plant Albizia Odoratissima,” Pharmaceutical Biology 55 (2017): 1155–1161. 28219296 10.1080/13880209.2017.1291694 PMC6130586

[22] M. O. Agbo , P. F. Uzor , U. N. Akazie‐Nneji , C. U. Eze‐Odurukwe , U. B. Ogbatue , and E. C. Mbaoji , “Antioxidant, Total Phenolic and Flavonoid Content of Selected Nigerian Medicinal Plants,” Dhaka University Journal of Pharmaceutical Sciences 14 (2015): 35–41.

[23] K. P. Bastola , Y. N. Guragain , V. Bhadriraju , and P. V. Vadlani , “Evaluation of Standards and Interfering Compounds in the Determination of Phenolics by Folin‐Ciocalteu Assay Method for Effective Bioprocessing of Biomass,” American Journal of American Chemistry 08 (2017): 416–431.

[24] C.‐C. Chang , M.‐H. Yang , H.‐M. Wen , and J.‐C. Chern , “Estimation of Total Flavonoid Content in Propolis by Two Complementary Colorimetric Methods,” Journal of Food and Drug Analysis 10 (2002): 178–182.

[25] N. Chaves , A. Santiago , and J. C. Alías , “Quantification of the Antioxidant Activity of Plant Extracts: Analysis of Sensitivity and Hierarchization Based on the Method Used,” Antioxidants 9 (2020): 76, 10.3390/antiox9010076. 31952329 PMC7023273

[26] A. Pokharel , A. Thapa , H. Karki , et al., “Chemical and Biological Analysis of Extracts of Acorus Calamus L,” Journal of Nepal Chemical Society 43 (2023): 151–158.

[27] M. Balouiri , M. Sadiki , and S. K. Ibnsouda , “Methods for in Vitro Evaluating Antimicrobial Activity: A Review,” Journal of Pharmaceutical Analysis 6 (2016): 71–79. 29403965 10.1016/j.jpha.2015.11.005 PMC5762448

[28] B. N. Meyer , N. R. Ferrigni , J. E. Putnam , L. B. Jacobsen , D. E. Nichols , and J. L. McLaughlin , “Brine Shrimp: A Convenient General Bioassay for Active Plant Constituents,” Planta Medica 45 (1982): 31–34. 7100305

[29] A. V. Krishnarajua , T. V. N. Raoa , M. Sundararajua , D. Vanisreeb , H.‐S. Tsayb , and A. G. V. S. Subbaraju , “Assessment of Bioactivity of Indian Medicinal Plants Using Brine Shrimp (Artemia salina) Lethality Assay”, International Journal of Applied Science and Engineering 3 2005: 125–134.

[30] Q. S. Sarah , F. C. Anny , and M. Misbahuddin , “Brine Shrimp Lethality Assay,” Bangladesh Journal of Pharmacology 12 (2017): 186–189, 10.3329/bjp.v12i2.32796.

[31] M. R. Hamidi , B. Jovanova , and T. K. Panovska , “Toxicological Evaluation of the Plant Products Using Brine Shrimp (Artemia salina L.) Model,” Maced Pharmaceutical Bulletin 60 (2014): 9–18, 10.33320/maced.pharm.bull.2014.60.01.002.

[32] S. M. A. Kawsar , M. Islam , S. Jesmin , M. A. Manchur , I. Hasan , and S. Rajia , “Evaluation of the Antimicrobial Activity and Cytotoxic Effect of some Uridine Derivatives”, International Journal of Biosciences 12 (2018): 211–219.

[33] B. Maharjan , L. Kumar Shrestha , J. P. Hill , et al., “Chemical Characterization of Corydalis Chaerophylla D.C. Extracts and Preliminary Evaluation of Their in Vitro and in Vivo Biological Properties,” Chemistry and Biodiversity 20, (2023): e202301209, 10.1002/cbdv.202301209. 37962402

[34] Y. Gang , T. Y. Eom , S. D. Marasinghe , Y. Lee , E. Jo , and C. Oh , “Optimizing the DPPH Assay for Cell‐free Marine Microorganism Supernatants,” Marine Drugs 19 (2021): 256, 10.3390/md19050256. 33947091 PMC8146261

[35] İ. Gulcin and S. H. Alwasel , “DPPH Radical Scavenging Assay,” Processes 11 (2023): 2248, 10.3390/pr11082248.

[36] N. S. N. M. Zin , N. A. S. Azmi , N. S. Anuar , et al., “A 96‐Well‐Plate–Based Method for the Estimation of Alpha‐Amylase Activity Using Miniaturises 3,5‐Dinitrosalicylic Acid (Dnsa) Colorimetric Method,” Malaysian Applied Biology 51 (2022): 95–102.

[37] R. Ranjitkar , D. P. Bhandari , and L. Bhandari , “Acute Toxicity Test of Ten Commercial Essential Oils of Nepalese Origin,” Journal of Plant Resources 17 (2019): 82–85.

[38] OECD , ‘Test Guideline 425: Acute Oral Toxicity—Up‐and‐Down Procedure’, Guidel Test Chem 2022, 26.

[39] D. Kumar , P. Sharma , K. Nepali , et al., “Antitumour, Acute Toxicity and Molecular Modeling Studies of 4‐(pyridin‐4‐yl)‐6‐(thiophen‐2‐yl) pyrimidin‐2(1H)‐one Against Ehrlich Ascites Carcinoma and Sarcoma‐180,” Heliyon 4 (2018): e00661. 30003157 10.1016/j.heliyon.2018.e00661 PMC6039700

